# Physical Fitness among Community-Dwelling Older Women with and without Risk of Falling—The Taipei Study, Taiwan

**DOI:** 10.3390/ijerph18147243

**Published:** 2021-07-06

**Authors:** Chen-Yi Song, Jau-Yih Tsauo, Pei-Hsin Fang, I-Yao Fang, Shao-Hsi Chang

**Affiliations:** 1 Department of Long-Term Care, National Taipei University of Nursing and Health Sciences, Taipei 112, Taiwan; 2 School and Graduate Institute of Physical Therapy, College of Medicine, National Taiwan University, Taipei 100, Taiwan; jytsauo@ntu.edu.tw; 3 Physical Education Center, Southern Taiwan University of Science and Technology, Tainan 710, Taiwan; peihsin@stust.edu.tw (P.-H.F.); fyy125@gmail.com (I.-Y.F.); 4 Department of Physical Education, National Taiwan Normal University, Taipei 106, Taiwan

**Keywords:** falls, fall risk, senior fitness test, elderly, aged

## Abstract

The purposes of this study were to compare the differences in physical fitness between community-dwelling older women fallers and non-fallers, with and without a risk of falling, and to investigate the relation between physical fitness and falling risk factors. This study was a secondary data analysis from a community- and exercise-based fall-prevention program. Baseline assessments pertaining to body weight and height, self-reported chronic diseases, the 12-item fall risk questionnaire (FRQ), senior fitness test, single-leg stand test, and handgrip strength test were extracted. Participants (*n* = 264) were classified into fallers and non-fallers, and sub-classified according to the risk of falling (FRQ ≥4 and <4). While controlling for the effect of age, body mass index (BMI), and multimorbidity, one-way analysis of covariance indicated that older women with a risk of falling showed poorer performances of the 8-foot up-and-go, 2-min step and 30-s chair stand compared with those without a risk of falling, regardless of the history of falls. Additionally, weaker grip strength was found in non-fallers with falling risk. Some significant, but low-to-moderate, correlations were found between physical fitness tests and fall risk factors in the FRQ, particularly in gait/balance problem and leg muscle weakness. Proactive efforts are encouraged to screen and manage deterioration in the identified physical fitness.

## 1. Introduction

The risk of falls is increased in older people, because of the age-related decline in physical function. Studies have indicated that poor physical function is related to a high risk of falls [1,2]. The literature has documented differences in muscle strength, endurance, agility, balance, and lower-extremity functional performance, between fallers and non-fallers, with fallers performing more poorly than non-fallers [3,4,5,6,7,8,9]. Among them, a multi-center study found a group difference in physical function tests when comparing fallers and non-fallers in the USA and Sweden, but not in Hong Kong [6], implying that the differences in physical function differed across countries.

Early identification of deficiencies in potential fall candidates (without falling experience) could help avoid the occurrence of falls. Functional fitness is an essential indicator of independence and quality of life in older adults in the late years, and it is also one of the most commonly reported indicators in fall-related research [10,11,12,13]. The handgrip strength test, as well as the single-leg stand test for static balance are frequently used in Asian countries and territories [9,14,15]. However, knowledge on the deficiencies in the physical fitness of potential fallers is limited. In terms of functional fitness, a study in Turkey used the Berg balance test for falling risk assessment in a sample of 60 elderly people living in a retirement home, and showed that the falling risk increases with the decline in upper- and lower-extremity muscle strength, aerobic endurance, agility and dynamic balance performance [11]. The study demonstrated the correlation between falling risk and functional fitness; however, the sample size was small, and the assessment of falling risk was balance-related. Another Hong Kong study compared functional fitness between community-dwelling older adults who were at risk of falling and those who were not, and demonstrated an overall reduced functional fitness capacity in older people with a risk of falling [10]. However, their results were limited by the small sample size (only 78 older adults) and narrow age span (65–74 years) of the study population recruited from a single community senior center. Additionally, the authors assessed falling risk by the Biodex balance system; this testing approach is not easy to administer or feasible in a community setting.

It is untenable to use balance-related measurement as a fall-risk test, because the etiology of falls among older people is complicated and multifactorial. The likelihood of falls increases with muscle weakness, unstable gait and/or balance, depression, vision impairment, fear of falling, and history of falls [16,17,18]. Hence, it would be more appropriate for a study to assess falling risk by a battery of risk factors. The fall risk questionnaire (FRQ) [19] certainly has a great potential to fulfill the needs. It incorporates the strongest evidence-based fall risk factors, has a strong agreement with clinical evaluation (kappa = 0.875, *p* < 0.0001), and demonstrates excellent sensitivity (100.0) and specificity (83.3), with a risk cut-off of ≥4 (ROC area under curve = 0.981) in a community-dwelling older adult population, offering a feasible way to identify older people who are at risk in community settings [19].

The Centers for Disease Control and Prevention developed the stopping elderly accidents, deaths, and injuries (STEADI) toolkit, based on the American Geriatrics Society/British Geriatrics Society (AGS/BGS) clinical practice guideline for the prevention of falls in older persons [20]. In the algorithm for fall risk assessment, falling history is a key question [21]. Older individuals who have fallen in the past year may be classified as low risk or moderate-to-high risk according to gait, strength, and balance evaluations. That is, fallers with no gait, strength, and balance problems would be classified as low risk. On the other hand, an individual without a falling history, but fears about falling or feels unsteady when standing and walking, may be at risk; an evaluation of gait, strength, and balance was needed for falling risk classification.

The purpose of this study was two-fold, as follows: First, to compare the differences in physical fitness between community-dwelling older fallers, and non-fallers with and without a risk of falling. Secondly, to investigate the relation between physical fitness and falling risk factors.

## 2. Materials and Methods

### 2.1. Study Design and Participants

This study was a secondary data analysis from a community- and exercise-based fall-prevention program conducted in 2017. Eligible participants meeting the following inclusion criteria: aged ≥65 years old, ambulant, pre-frail or frail (defined by SOF frailty index [22]) or had history of falls in the past year, and absence of dementia were identified by 12 district health centers of Taipei City, and were invited to participate. Seventy-three men and 264 women participated in the fall-prevention program. The Institutional Review Board of National Taiwan Normal University approved the study, and all participants provided signed informed consent prior to participation.

Baseline assessments pertaining to body weight and height, self-reported chronic diseases (e.g., hypertension, diabetes, and coronary arterial disease), fall risk and physical fitness were extracted. The study only targeted women because female sex is a risk factor significantly associated with fall and fall-related injury among older Chinese people [23,24]. 

### 2.2. Fall Risk Assessment

In this study, a fall refers to an event that results in a person coming to rest inadvertently on the ground or floor or other lower level. The 12-item fall risk questionnaire (FRQ) was used to evaluate risk factors associated with falls, such as history of falls, fear of falling, gait and balance problems, leg muscle weakness, impaired sensation and proprioception, depression, and side effects of medications [12,19]. It was developed based on evidence and has clinical acceptability, and it was found to have good concurrent validity. The cut-off score for falling risk is ≥4 [19]. Therefore, participants with an FRQ score of 4 or greater had a high risk of falling.

### 2.3. Physical Fitness Assessment

The senior fitness test was used to evaluate physical fitness. It includes a 30-s chair stand test for assessing lower-limb muscle strength, a 30-s arm curl test for assessing upper-limb muscle strength, a 2-min step test for evaluating aerobic endurance, a chair sit-and-reach test for evaluating lower-body flexibility, a back scratch test for investigating upper-body flexibility, and an 8-foot up-and-go test for examining agility and dynamic balance. Participants were scheduled for assessment in accordance with their living district. All tests were performed on the same day by trained research assistants in the local community center. The procedure outlined in the test manual was followed [25].

In addition, a single-leg stand test was administered to evaluate static balance [15]. Participants stood on their dominant leg, and held the one-legged stance while keeping their hands on their waist and their foot lifted off the floor. The test was terminated following a maximum of 30 s, and the best score of two testing trials was used for analysis.

The handgrip strength of participants’ dominant hands was assessed using a hand dynamometer (TTM-YD, Tokyo, Japan). Participants were asked to stand with their arms by the side of the body and were given verbal encouragement to give their maximum effort [12]. The best value of two trials with 1-min breaks was taken.

### 2.4. Statistical Analyses

Statistical analyses were performed using SPSS 22.0 for Windows (IBM Corp; Armonk, NY, USA). Descriptive statistics were calculated to analyze participants’ demographics. Participants were classified into fallers and non-fallers (defined as those with and without a history of falls in the past year, respectively), and sub-classified according to risk of falling (FRQ ≥4 and <4). Due to group differences in age, body mass index (BMI) and multimorbidity, the one-way analysis of covariance (ANCOVA) was applied to compare differences of physical fitness variables between community-dwelling older women fallers and non-fallers with and without a risk of falling. The *p*-value was adjusted (0.006) for multiple comparisons. The LSD post hoc testing was performed for multiple pairwise comparisons. A nonparametric partial correlation was used to investigate relationships between physical fitness variables and risk factors associated with falls in the FRQ while controlling for age, BMI and multimorbidity. A correlation coefficient of 0.1 represents a “weak” association, whereas 0.4 is a “moderate” association, and 0.7 is a “strong” association [26].

## 3. Results

### 3.1. General Characteristics

The basic characteristics of the participants are shown in Table 1. The mean age of the 264 participants were 74.8 ± 6.7 years (ranging from 65 to 95 years). In total, 105 participants (39.8%) experienced falls in the past year, with a mean FRQ score of 5.4 ± 2.8, whereas 159 participants (60.2%) had no fall history in the past year, showing a mean FRQ score of 2.3 ± 2.6. Among the fallers and non-fallers, 69 (65.7%) and 41 participants (25.8%) were at risk of falling, and their mean FRQ scores were 6.9 ± 2.3 and 6.1 ± 1.0 compared with 2.5 ± 0.5 and 0.9 ± 1.0 for those without a risk of falling, respectively. The age, BMI and multimorbidity were different between the groups.

### 3.2. Comparison of Physical Fitness According to the Experience of Falls and the Risk of Falling

Table 2 shows the differences in physical fitness between community-dwelling older women fallers and non-fallers with and without a risk of falling. Significant differences were observed in the performances of 8-foot up-and-go, 30-s chair stand, 2-min step, and handgrip strength (all *p* < 0.006). Compared with those without a risk of falling, older women with a risk of falling showed poorer 8-foot up-and-go, 2-min step and 30-s chair stand performances. Older women non-fallers with a risk of falling had weaker handgrip strength compared with those without a risk of falling.

### 3.3. Relationship between Physical Fitness and Fall Risk Factors

Some significant, but low-to-moderate, correlations were found between physical fitness tests and fall risk factors in the FRQ, particularly in gait/balance problem and leg muscle weakness, with adjustments for age, BMI and multimorbidity (Table 3).

## 4. Discussion

This study demonstrated differences in physical fitness among older Taiwanese women with and without a risk of falling, after other variates were adjusted. The older women with a risk of falling exhibited a significantly poorer performance of the 8-foot up-and-go, 2-min step and 30-s chair stand than those without a risk of falling did. The at-risk non-fallers also had weaker handgrip strength compared with those without a risk of falling.

Agility and dynamic balance are the parameters most likely to deteriorate earlier, and are the most relevant to the risk of falling [10,11]. In this study, community-dwelling older women with a risk of falling took 8.3 s, on average, to complete the up-and-go test. Similarly, a previous study indicated that a cut-off score of 8.5 s for the up-and-go test could predict which older adults were most likely to be fallers [27]. Additionally, we found that the at-risk women’s number of chair stands was 3–5 less than the national norms of Taiwan [14], thus supporting the reduction in lower-extremity muscle strength to be one of the main determinants of falls in older people [11]. In addition to agility and lower-extremity strength, aerobic endurance was reported as a key indicator in discriminating older adults with and without a fall risk [10]. The between-group differences in the 2-min step test observed in this study (80.3 vs. 97.6) were similar to those in a Hong Kong study (82.7 vs. 92.3) [10]. As good balance control and leg strength are required for this test, those who have more difficulty in performing the 2-min step test would be more prone to falling.

Older women with fall risk demonstrated subtle balance impairments during single-leg standing; the performance was less than the normative values [28]. This result is not surprising, considering that the duration of the single-leg stance was reported to be significantly related to leg strength, aerobic endurance, agility, and dynamic balance [11].

Previous studies found that fallers performed inferior in the handgrip strength test compared to non-fallers in the USA, Sweden [6], Australia [29], as well as Korea and Taiwan’s elderly populations [7,9]. Our study further demonstrated that non-fallers with a falling risk had weaker handgrip strength. Together with the study’s findings that handgrip strength could predict future falls [30,31], the handgrip strength test could be considered as a simple and inexpensive test for identifying potential fallers in community settings.

The significance of age, BMI and multimorbidity found between older women with and without falling risk in this study may be expected. Age is one of the key risk factors for falls [23,29,32]. Using Asian BMI classification, a BMI ≥25 appears to be associated with a greater risk of falling in older adults [33]. Chronic diseases have a large impact on both the risk of falls and their occurrence [29]. Even after accounting for these potential confounders, we were able to show that at-risk women performed inferior in the 8-foot up-and-go, 2-min step, 30-s chair stand, and handgrip strength tests. Additionally, several significant correlations were found between the physical fitness tests and fall risk factors in the FRQ, especially gait/balance problem and leg muscle weakness. However, the overall associations were weak. Only one moderate correlation was shown between the 8-foot up-and-go and the FRQ item “I use or have been advised to use a cane or walker to get around safely.” Our finding was consistent with the result of a previous study that found a substantial agreement between the assistive device item and the Tinetti performance-oriented mobility assessment-gait (kappa = 0.698, *p* < 0.0001) [19]. Another study also found moderate correlations between the Berg balance test (BBT) and the 8-foot up-and-go, 2-min step, and 30-s chair stand tests, but no correlation was found between the BBT and the chair sit-and-reach test and back scratch test [11]. Although two Korean studies have found correlations between a fear of falling and physical performance, regardless of the experience of falls [7,34], this study found no such correlation. This can partly be explained by the significant correlations found between the fear of falling, physical performance, age, and BMI [7]. Furthermore, the fear of falling was assessed by a single item in the current study, but by a questionnaire (fall efficacy scale) in the Korean studies.

This study is the first to comprehensively explore the falling risk and physical fitness, including functional fitness and handgrip strength, in community-dwelling older women fallers and non-fallers. The strength of this study was that functional fitness and handgrip strength were feasible measures for conducting in the community, which can easily be replicated in other countries for the early identification of potential fall candidates. However, there were several limitations. First, because the study participants were female, the generalizability of the findings is limited. Second, while the FRQ was used in Chinese studies for fall risk assessment [12,35], the questionnaire has not been validated in the Chinese population. Third, our study did not include a measurement of the participants’ lifestyle and physical activity, which could influence their fitness status and the occurrence of falls, which compromises our results [6,9,29]. Furthermore, the causal relationships between physical fitness and future falls needs to be confirmed in prospective studies. Finally, future research could investigate functional fitness profiles among community-residing older adults with different fall risk phenotypes.

## 5. Conclusions

Older Taiwanese women with a falling risk were found to have poorer 8-foot up-and-go, 2-min step and 30-s chair stand performances. Additionally, weaker grip strength was found in non-fallers with a falling risk. Proactive efforts are encouraged to screen and manage deterioration in the identified physical fitness.

## Figures and Tables

**Table 1:** Basic characteristics of the participants.

	Nonfallers		Fallers			
Risk of Falling	No (*n* = 118) Group A	Yes (*n* = 41) Group B	No (*n* = 36) Group C	Yes (*n* = 69) Group D	*p*	Post Hoc Comparison
Age, year	73.0 ± 5.3	79.8 ± 7.0	72.6 ± 5.8	76.0 ± 7.2	<0.001 *	Group A < B, D Group C < B, D Group D < B
Height, m	153.1 ± 5.5	152.0 ± 6.0	152.8 ± 6.4	150.9 ± 5.9	0.106	
Weight, kg	54.7 ± 8.8	59.0 ± 10.7	57.3 ± 7.5	55.4 ± 9.5	0.062	
BMI, kg/m^2^	23.33 ± 3.47	25.59 ± 4.91	24.42 ± 3.18	24.30 ± 4.12	0.013 *	Group A < B
Multimorbidity	0.9 ± 1.0	2.0 ± 1.1	1.1 ± 0.8	1.6 ± 1.3	<0.001 *	Group A < B, D Group C < B, D

* *p* < 0.05.

**Table 2:** Comparisons of physical fitness between community-dwelling elderly women fallers and non-fallers with and without a risk of falling.

	Non-Fallers		Fallers			
Risk of Falling	No (*n* = 118) Group A	Yes (*n* = 41) Group B	No (*n* = 36) Group C	Yes (*n* = 69) Group D	*p*	Post Hoc Comparison
Chair sit and reach, cm	8.17 ± 9.53	3.89 ± 11.27	8.22 ± 10.02	5.21 ± 12.14	0.449	
Back scratch, cm	1.80 ± 11.40	−4.60 ± 13.71	4.23 ± 9.51	2.05 ± 10.90	0.032	
8-ft up-and-go, s	6.20 ± 1.31	8.88 ± 3.44	6.11 ± 1.39	7.98 ± 3.00	<0.001 *	Group A < B, D Group C < B, D
Chair stand, no.	19.0 ± 5.3	14.1 ± 5.9	18.4 ± 3.9	15.9 ± 4.9	<0.001 *	Group A > B, D Group C > B *(p* = 0.008*)*, D (*p* = 0.010)
Arm curl, no.	17.0 ± 4.2	14.0 ± 4.5	16.6 ± 3.9	15.1 ± 4.1	0.024	
2-min step, no.	97.2 ± 19.3	76.3 ± 22.4	98.8 ± 17.0	82.4 ± 27.1	<0.001 *	Group A > B, D Group C > B, D
Single-leg stand, s	17.14 ± 11.08	11.35 ± 11.83	20.25 ± 10.04	14.13 ± 11.27	0.021	
Handgrip strength, kg	22.50 ± 4.62	19.77 ± 4.93	23.79 ± 3.99	21.26 ± 4.29	0.001 *	Group A > B Group C > B

* *p* < 0.006.

**Table 3:** Correlations between the fall risk questionnaire items and physical fitness.

Variable	Chair Sit and Reach	Back Scratch	8-Ft Up and Go	Chair Stand	Arm Curl	2-Min Step	Single Leg Stand	Handgrip Strength
History of previous falls	−0.053	0.173 *	0.044	−0.140 *	−0.096	−0.062	0.044	0.038
Can or walker use	−0.059	0.071	0.441 *	−0.203 *	−0.191 *	−0.283 *	−0.059	−0.186 *
Unsteady gait	0.043	0.024	0.159 *	−0.141 *	−0.145 *	−0.166 *	−0.048	−0.010
Poor balance	−0.194 *	−0.020	0.221 *	−0.164 *	−0.091	−0.194 *	0.084	−0.255 *
Fear of falling	0.052	−0.073	0.037	−0.035	−0.091	−0.002	0.008	−0.053
Leg muscle weakness (stand up from a chair)	−0.056	0.038	0.205 *	−0.205 *	−0.200 *	−0.167 *	0.036	−0.152 *
Leg muscle weakness (stepping up onto a curb)	0.006	−0.031	0.205 *	−0.188 *	−0.191 *	−0.125	−0.110	−0.046
Incontinence	−0.063	−0.010	0.034	−0.066	−0.086	−0.018	0.008	0.003
Impaired sensation and proprioception	−0.028	−0.074	−0.030	0.065	−0.062	−0.018	0.048	−0.053
Side effects of medications	−0.021	−0.058	0.018	−0.141 *	−0.111	−0.039	−0.024	−0.027
Sleep/mood medications	−0.054	−0.056	0.052	−0.089	−0.064	−0.003	0.120	−0.145 *
Depression	−0.051	−0.010	0.052	−0.036	−0.104	−0.074	0.026	−0.078

* *p* < 0.05.

## Data Availability

The datasets used and/or analyzed during the current study are available from the corresponding authors on reasonable request.
